# IMPACT OF HLA-G IN THE OUTCOME OF VITILIGO IN TUNISIAN PATIENTS

**DOI:** 10.4103/0019-5154.60346

**Published:** 2010

**Authors:** Akrem Jalel, Aouadi Ridha, Duboisier Laurent, Moureaux Philippe, M H Hamdaoui

**Affiliations:** 1 *From the Research Unit on the Antioxidant Compounds, Oxidative Stress, Trace Elements and Metabolic Diseases, Department of Physiology, Ecole Supérieure des Sciences et Techniques de la Santé de Tunis, Tunisia*; 2 *From the Department of Dermatological and Venereal Diseases Hospital Foschi, Suresnes, Paris*; 3 *From the Laboratory of Immunogenetics, Department of Immunology Research, France.*

**Keywords:** *Vitiligo*, *HLA-G*, *immune tolerogenesis*

## Abstract

**Background:**

The human leukocyte antigen (HLA) system in the skin coordinates the pigmentation and immune response and could be implicated in the pathogenesis of vitiligo. Human leukocyte antigen HLA-G is a nonclassic, major histocompatibility complex class I molecule expressed in the extravillous cytotrophoblast at the feto-maternal interface. It is known to protect the fetus from maternal cellular immunity. Analogically, it could be implicated in the pathogenesis of autoimmune diseases such as vitiligo.

**Aims:**

To compare the expression of HLA-G between vitiligo patients and healthy controls.

**Materials and Methods:**

In the present study, 22 vitiligo patients and 24 healthy controls were investigated to look for a possible correlation between HLA-G expression and this pathology. Expression of HLA-G in cutaneous biopsy specimens was investigated by immunohistochemical analysis.

**Results:**

HLA-G was detected in the biopsy specimens of 3 (13%) out of 22 patients. This number was significantly higher in healthy controls 18 (75%) out of 24 as compared to vitiligo patients (*P* < 0.001).

**Conclusion:**

There is significant negative correlation between HLA-G expression and vitiligo. In our mind, upregulation of HLA-G expression in lesional skin could be local (superficial expression) or systemic (soluble HLA-G isoforms) compensation to restore normal pigmentation in lesions.

## Introduction

Vitiligo is an acquired cutaneous disorder that presents with gradual skin depigmentation produced by the deterioration of melanocyte functions. Several hypotheses have been proposed to explain the dysfunction and/or loss of melanocytes in the epidermis of vitiligo patients.[1,2] These include an autoimmune mechanism, an auto-cytotoxic mechanism, and an abnormality in the melanocytes or in the surrounding keratinocytes producing factors, necessary for the survival and function of melanocytes.[3–5] Until now, the pathogenesis of vitiligo remains partially understood and probably involves various combinations of diverse mechanisms. The nonclassic human leukocyte antigen (HLA) class I molecule HLA-G, expressed on extravillous cytotrophoblasts at the feto-maternal interface during pregnancy,[6,7] has been reported to play a role in mediating maternal tolerance of the fetal “semiallogeneic” graft.[8] This HLA-G antigen exhibits low polymorphism and can be expressed as both membrane-bound proteins (HLA-G1, HLA-G2, HLA-G3, HLA-G4) and soluble isoforms (HLA-G5, HLA-G6, HLA-G7).[9,10] The soluble isoforms HLA-G5 and HLA-G6 have been detected in the amniotic fluid and serum of pregnant women.[11,12] HLA-G expression was initially found to be restricted to the placenta. More recently, its expression has been detected in thymic epithelial cells,[13] various malignant cells,[14–16] and in peripheral blood monocytes, activated by IL-10.[17] HLA-G is known to inhibit the cytotoxic activity of T lymphocytes and natural killer cells (NK),[18,19] which are essential effector cells in a melanocyte attack.[20] The purpose of the present study is to examine HLA-G expression in the lesional skin of vitiligo patients and in the skin of healthy controls.

## Materials and Methods

The study protocols and informed consent forms were approved by the Ethical Review Committee of the Medenine Hospital Centre (large area in the south of Tunisia). Informed consent was obtained from all participants. The patients and control subjects in the study were Tunisians. Unrelated patients with vitiligo (*n* = 22; 10 females; 12 males; age range 22-75 years) were included in the study. The mean age of vitiligo onset of the patients was 32.5 years and the mean duration of vitiligo was 16.5 years. Five patients had a family history of vitiligo. None of the patients included in the study had received any specific therapy in the previous three months. The clinical signs on which the diagnosis of vitiligo was based were characteristic loss of skin pigmentation with typical localization and white color on the skin lesions under Wood's lamp. The type of vitiligo was based on the extent of involvement and the distribution of pigmentation. The control group consisted of healthy volunteers (*n* = 24; 7 females; 17 males; age range 21 to 67 years) with no family history of vitiligo or other chronic dermatoses. One skin biopsy (Ø 3.5 mm) was obtained from each patient with vitiligo: One from the central part of the involved skin. Another skin biopsy (Ø 3.5 mm) was taken from the skin of healthy control subjects. All probands had skin phototype II (8 controls, 13 patients) or III (16 controls, 9 patients), Fitzpatrick classification. The biopsies were instantaneously stored at −80°C until further use.

### Laboratory investigation

Expression of the HLA-G molecule was analyzed by the immunohistochemical analysis of the cutaneous biopsy specimens. In addition to the biopsy specimens used for histopathological analysis and immunohistochemistry, control specimens were obtained from two human trophoblasts as a positive control. Monoclonal and polyclonal antibodies were used for the detection of HLA-G molecules in the biopsy specimens and serum of patients. monoclonal antibodies (mAbs) used were 87G IgG2a anti-HLA-G1 and -G5 (provided by D. Geraghty, Fred Hutchinson Cancer Research, Seattle, Washington), 4H84 IgG1 anti-denatured HLA-G heavy chain (provided by M. McMaster, University of California, San Francisco), and W6/32 IgG2a anti-HLA class I heavy chains associated with β2m (Sigma, Milwaukee, Wisconsin). An isotype-matched antibody (Sigma) was used as the control. A rabbit polyclonal antibody PAG5-6 generated against the C-terminal peptide of the HLA-G α-chain encoded by intron 4 sequences was used to specifically recognize the soluble forms HLA-G5 and HLA-G6.[21]

### Histology and immunohistochemistry

For histology, 4-μm-thick sections were obtained from each paraffin block and stained with hematoxylin and eosin. For immunohistochemical studies, 6-μm-thick sections of frozen tissues were fixed for 10 minutes in cold acetone, dehydrated, and permeabilized with saponine in phosphate buffered saline (PBS). Staining procedures were processed with the Dako Envision System (DAKO). Samples were incubated for 30 minutes in 50% human normal serum, in PBS, to eliminate nonspecific bindings. The samples were incubated with the following primary mAbs for 30 minutes: W6/32, 87G, 4H84 mAbs, and control antibody and followed by incubation with a secondary conjugated goat anti-mouse/antibody coupled with peroxidase (DAKO) for 30 minutes. After incubation for 10 minutes with a substrate, sections were counterstained with hematoxylin dye and mounted with antimounting medium (DAKO).

### Statistical analysis

Data are presented as mean ± SEM. The Student's *t* test was used, and a value of *P* < 0.05 was considered significant.

## Results

### Histopathology

**Figure 1:**
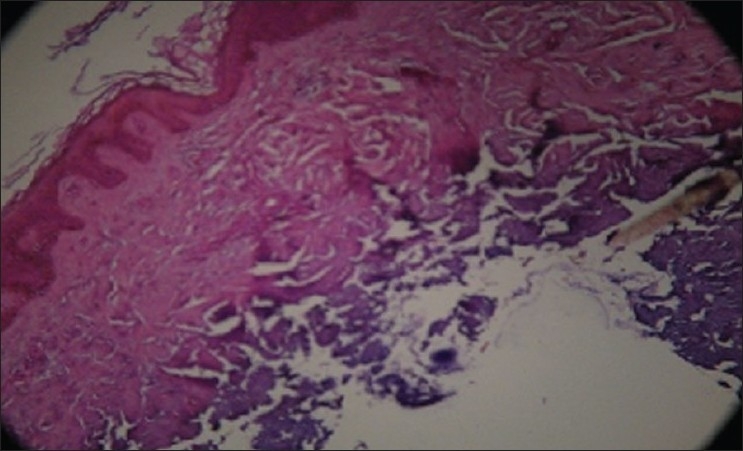
Low magnification (10×) with Haematoxylin eosin, conservation of the basal layer, intense pigmentation and presence of melanin granules

**Figure 2:**
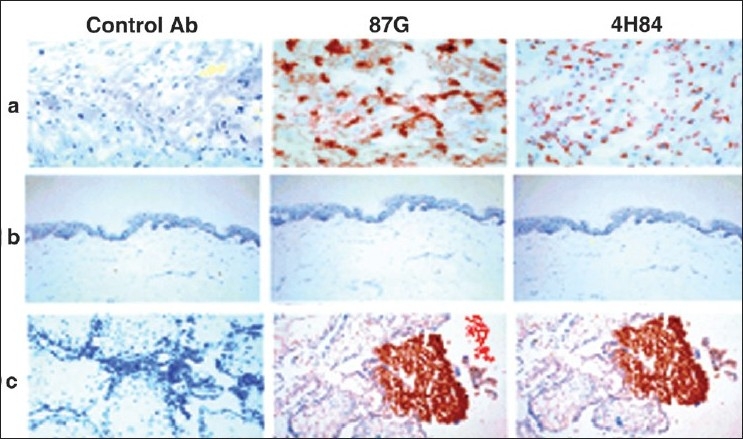
Immunohistochemical analysis of HLA-G expression in cutaneous biopsy samples (a, b), trophoblast as positive control (c). Control antibody 87G detects HLA-G1 and HLA-G5 isoforms, and 4H84 detects the denatured form of HLA-G. a) Vitiliginous cutaneous biopsy specimen: positive staining with both 87G and 4H84; b) Control biopsy specimen: Negative staining with 87G and positive staining with 4H84; c) Trophoblast biopsy specimen: Positive staining with both 87G and 4H84.

For histology, 4-μm-thick sections were obtained from each paraffin block and stained with hematoxylin and eosin [Figures 1 and 2].

### Expression of HLA-G in cutaneous biopsy specimens

Expression of HLA-G antigens in sections of vitiliginous cutaneous biopsy specimens was investigated through immunohistochemical analysis with the use of HLA-G mAbs: 87G IgG2a specific for HLA-G1 and HLA-G5 and 4H84 IgG1, which recognizes the α-1 domain (pan-HLA-G). Staining with W6/32 IgG2a was used to control the presence of intact HLA class I molecules in different tissues and was positive in all tested tissue biopsy specimens. We detected HLA-G proteins in 3 vitiligo patients. One patient specimen was positively stained by both 87G and 4H84 mAbs and two were positively stained by 4H84 mAb only. Trophoblasts used as positive controls exhibited strong staining with 87G and 4H84 mAbs, and skin biopsy specimens taken from healthy controls were also positively stained by 87G and 4H84 mAb.

## Discussion

In the skin, the potential role of the HLA system in pigmentation is not yet clarified, it's possible role in the pathogenesis of vitiligo as a depigmentation disorder remains unclear. The development of tolerance requires that the immune system utilize several strategies for neutralizing self-reactive T cells. These strategies include deletion,[22] anergy,[23] and immunoregulatory pathways.[24] This study suggests that the expression of HLA-G may be an alternative strategy to downregulate the acute immunoresponse against melanocytes. *In vitro*, soluble HLA-G has been demonstrated to induce apoptosis of activated CD8^+^ T cells[25] and to modulate NK[26] and allo-CTL response,[27] whereas, membrane-bound HLA-G proteins have been shown to inhibit both NK and T cell-mediated cytolysis,[8,20] to suppress the proliferation of allospecific CD4^+^ T lymphocytes,^[28]^ and to induce Th2 cytokine profile.[29] Interestingly, Graham *and al*., recently demonstrated that soluble HLA-G protein secreted by allo-specific CD4^+^ T cells suppresses the allo-proliferative response.[30] In the present study, HLA-G expression was found in 13% of the 22 vitiligo patients. There was no case of extensive vitiligo between HLA-G-positive vitiligo patients. No significant difference between age, race, or sex could be detected between HLA-G-positive and HLA-G-negative patients. Therefore, the expression of HLA-G was likely deactivated during the process of vitiligo. It is possible that the suppression of melanin production deactivates HLA-G transcriptionally through negative feedback, to restore normal pigmentation. In melanocyte graft, HLA-G expression may allow escape from recognition and destruction of the melanocytes by alloreactive T cells. Furthermore, the soluble HLA-G forms, HLA-G5 and HLA-G6, may play an additional role in inhibiting the cytotoxic activity of NK cells.[31] Experimentally, numerous factors have been shown to upregulate HLA-G expression, such as IL-10.[32] An IL-10-HLA-G autocrine effect may contribute to melanocyte graft tolerance. However, we cannot exclude that HLA-G-positive patients may carry out specific HLA-G alleles associated with high HLA-G production.[33] The search for HLA-G expression in vitiligo patients could provide a new understanding of the factors implicated in vitiligo and a new modulation of immunosuppressive therapy in this pathology. For example, the use of soluble forms of HLA-G could contribute to immunosuppression and make it possible to reduce the amount of other immunosuppressive agents (corticoids and immunomodulators). The present study supports the hypothesis that negative HLA-G expression is associated with vitiligo. HLA-G expression seems to minimize active vitiligo. It remains to be understood why some vitiligo patients express HLA-G, whereas, other negative controls didn't develop vitiligo.

## References

[1] Tobin DJ, Swanson NN, Pittelkow MR, Peters EM, Schallreuter KU (2000). Melanocytes are not absent in lesional skin of long duration vitiligo. J Pathol.

[2] Le Poole IC, Das PK, van den Wijngaard RM, Bos JD, Westerhof W (1993). Review of the etiopathomechanism of vitiligo: A convergence theory. Exp Dermatol.

[3] Ongenae K, van Geel N, Naeyaert JM (2003). Evidence for an autoimmune pathogenesis of vitiligo. Pigm Cell Res.

[4] Schallreuter KU, Moore J, Wood JM, Beazley WD, Gaze DC, Tobin DJ (1999). *In vivo* and *in vitro* evidence for hydrogen peroxide (H_2_ O_2_) accumulation in the epidermis of patients with vitiligo and its successful removal by a UVB-activated pseudocatalase. J Investig Dermatol Symp Proc.

[5] Moretti S, Spallanzani A, Amato L, Hautmann G, Gallerani I, Fabiani M (2002). New insights into the pathogenesis of vitiligo: Imbalance of epidermal cytokines at sites of lesions. Pigm Cell Res.

[6] Kovats S, Main EK, Librach C, Stubblebine M, Fisher SJ, DeMars R (1990). A class I antigen HLA-G expressed in human trophoblasts. Science.

[7] McMaster MT, Librach CL, Zhou Y, Lim KH, Janatpour MJ, DeMars R (1995). Human placental HLA-G expression is restricted to differentiated cytotrophoblasts. J Immunol.

[8] Rouas-Freiss N, Gonçalves RM, Menier C, Dausset J, Carosella ED (1997). Direct evidence to support the role of HLA-G in protecting the fetus from maternal uterine natural killer cytolysis. Proc Natl Acad Sci USA.

[9] Ishitani AG, Geraghty DE (1992). Alternative splicing of HLA-G transcripts yields proteins with primary structures resembling both class I and class II antigens. Proc Natl Acad Sci USA.

[10] Fujii T, Ishitani A, Gerghty DE (1994). A soluble form of HLA-G antigen is encoded by a messenger ribonucleic acid containing intron 4. J Immunol.

[11] Hunt JS, Jadhav L, Chu W, Geraghty DE, Ober C (2000). Soluble HLA-G circulates in maternal blood during pregnancy. Am J Obstet Gyneocol.

[12] Rebmann V, Pfeiffer K, Passler M, Ferrone S, Maier S, Weiss E (1999). Detection of soluble HLA-G molecules in plasma and amniotic fluid. Tissue Antigens.

[13] Crisa L, McMaster MT, Ishii JF, Fisher SJ, Salomon DR (1997). Identification of a thymic epithelial cell subset sharing expression of the class Ib HLA-G molecule with fetal trophoblasts. J Exp Med.

[14] Paul P, Cabestré FA, Le Gal FA, Khalil-Daher I, Le Danff C, Schmid M (1999). Heterogeneity of HLA-G gene transcription and protein expression in malignant melanoma biopsies. Cancer Res.

[15] Paul P, Rouas-Freiss N, Khalil-Daher I, Moreau P, Riteau B, Le Gal FA (1998). HLA-G expression in melanoma: A way for tumor cells to escape from immunosurveillance. Proc Natl Acad Sci USA.

[16] Wagner SN, Rebmann V, Willers CP, Grosse-Wilde H, Goos M (2000). Expression analysis of classic and non-classic HLA molecules before interferon alfa-2b treatment of melanoma. Lancet.

[17] Moreau P, Adrian-Cabestre F, Menier C, Guiard V, Gourand L, Dausset J (1999). IL10 selectively induces HLA-G expression in human trophoblasts and monocytes. Int Immunol.

[18] Rouas-Freiss N, Marchal RE, Kirszenbaum M, Dausset J, Carosella ED (1997). The alpha 1 domain of HLA-G1 and HLA-G2 inhibits cytotoxicity induced by natural killer cell: Is HLA-G the public ligand for natural killer inhibitory receptors?. Proc Natl Acad Sci USA.

[19] Riteau B, Rouas-Freiss N, Menier C, Paul P, Dausset J, Carosella ED (2001). HLA-G2, -G3 and -G4 isoforms expressed as nonmature cell surface glycoproteins inhibit NK antigen-specific CTL cytolysis. J Immunol.

[20] Lila N, Carpentier A, Amrein C, Khalil-Daher I, Dausset J, Carosella ED (2000). Implication of HLA-G molecule in heart-graft acceptance. Lancet.

[21] Paul P, Cabestre FA, Ibrahim EC, Lefebvre S, Khalil-Daher I, Vazeux G (2000). Identification of HLA-G7 as a new splice variant of HLA-G mRNA and expression of soluble HLA-G5, -G6, and -G7 transcripts in human transfected cells. Hum Immunol.

[22] Nikolic B, Sykes M (1996). Clonal deletion as a mechanism of transplantation tolerance. J Heart Lung Transplant.

[23] Rocha B, Tanchot C, Von Boehmer H (1993). Clonal energy blocks *in vivo* growth of mature T cells and can be reserved in the absence of antigen. J Exp Med.

[24] Davies JD, Martin G, Phillips J, Marshall SE, Cobbold SP, Waldmann H (1996). T cell regulation in adult transplantation tolerance. J Immunol.

[25] Fournel S, Aguerre-Girr M, Huc X, Lenfant F, Alam A, Toubert A (2000). Soluble HLA-G1 triggers CD95/CD95 ligand-mediated apoptosis in activated CD8+ cells by interacting with CD8. J Immunol.

[26] Marchal-Bras Concalves R, Rouas-Freiss N, Connan F, Choppin J, Dausset J, Carosella ED (2001). A soluble HLA-G protein that inhibits natural killer cell-mediated cytotoxicity. Transplant Proc.

[27] Kapasi K, Albert SE, Yie SM, Zavazava N, Librach CL (2000). HLA-G has a concentration-dependent effect on the generation of an allo-CTL response. Immunology.

[28] Bainbridge D, Ellis SA, Sargent IL (2000). HLA-G suppresses proliferation of CD4+ T-lymphocytes. J Reprod Immunol.

[29] Kanai T, Fujii T, Kozuma S, Yamashita T, Miki A, Kikuchi A (2001). Soluble HLA-G influences the release of cytokines from allogeneic peripheral blood mononuclear cells in culture. Hum Reprod.

[30] Lila N, Rouas-Freiss N, Dausset J, Carpentier A, Carosella ED (2001). Soluble HLA-G protein secreted by allo-specific CD41 T cells suppresses the allo-proliferative response: ACD41 T cell regulatory mechanism. Proc Natl Acad Sci USA.

[31] Rebmann V, van Der Ven K, Pabler M, Pfeiffer K, Krebs D, Grosse-Wilde H (2001). Association of soluble HLA-G plasma levels with HLA-G alleles. Tissue Antigens.

[32] Thody AJ, Ridley K, Penny RJ, Chalmers R, Fisher C, Shuster S (1983). MSH peptides are present in mammalian skin. Peptides.

[33] Pichler R, Sfetsos K, Badics B, Gutenbrunner S, Aubock J (2006). Vitiligo patients present lower plasma levels of alpha-melanotropin immunoreactivities. Neuropeptides.

